# Block Aligner: an adaptive SIMD-accelerated aligner for sequences and position-specific scoring matrices

**DOI:** 10.1093/bioinformatics/btad487

**Published:** 2023-08-03

**Authors:** Daniel Liu, Martin Steinegger

**Affiliations:** University of California Los Angeles, Los Angeles, CA, United States; School of Biological Sciences, Artificial Intelligence Institute, Institute of Molecular Biology and Genetics, Seoul National University, Seoul, South Korea

## Abstract

**Motivation:**

Efficiently aligning sequences is a fundamental problem in bioinformatics. Many recent algorithms for computing alignments through Smith–Waterman–Gotoh dynamic programming (DP) exploit Single Instruction Multiple Data (SIMD) operations on modern CPUs for speed. However, these advances have largely ignored difficulties associated with efficiently handling complex scoring matrices or large gaps (insertions or deletions).

**Results:**

We propose a new SIMD-accelerated algorithm called Block Aligner for aligning nucleotide and protein sequences against other sequences or position-specific scoring matrices. We introduce a new paradigm that uses blocks in the DP matrix that greedily shift, grow, and shrink. This approach allows regions of the DP matrix to be adaptively computed. Our algorithm reaches over 5–10 times faster than some previous methods while incurring an error rate of less than 3% on protein and long read datasets, despite large gaps and low sequence identities.

**Availability and implementation:**

Our algorithm is implemented for global, local, and *X*-drop alignments. It is available as a Rust library (with C bindings) at https://github.com/Daniel-Liu-c0deb0t/block-aligner.

## 1 Introduction

Efficiently aligning biological sequences is an incredibly important problem due to its prevalence in many bioinformatics workflows and the ever-increasing scale of experiments. For example, protein sequences are aligned in large-scale database searches (Mirdita *et al.* 2017, Steinegger and Söding 2017). These alignments typically use the BLOSUM (Henikoff and Henikoff 1992) scoring matrices to capture the similarity between different amino acids. To increase the sensitivity of database searches and detect distantly related proteins, queries also make use of position-specific scoring matrix (PSSM) profiles that describe an alignment of multiple sequences (Altschul *et al.* 1997, Steinegger and Söding 2017). PSSMs are also used during the construction of multiple sequence alignments (Thompson *et al.* 1994, Katoh *et al.* 2002). On the other hand, in DNA or RNA sequencing experiments, reads of varying lengths are compared with each other or aligned to reference assemblies (Langmead and Salzberg 2012, Li 2013, 2018). For these use cases, the alignment algorithm must tolerate sequencing errors and reveal genetic variations (Alser *et al.* 2021, Sahlin *et al.* 2022). The classic algorithm used for computing optimal pairwise alignment (with affine gap penalties) through 2D dynamic programming (DP) is the Smith–Waterman–Gotoh algorithm (Gotoh 1982), and its global variant (Needleman and Wunsch 1970). However, this is very slow, as its runtime and memory usage scales quadratically for increasing sequence lengths.

**Figure 1.**
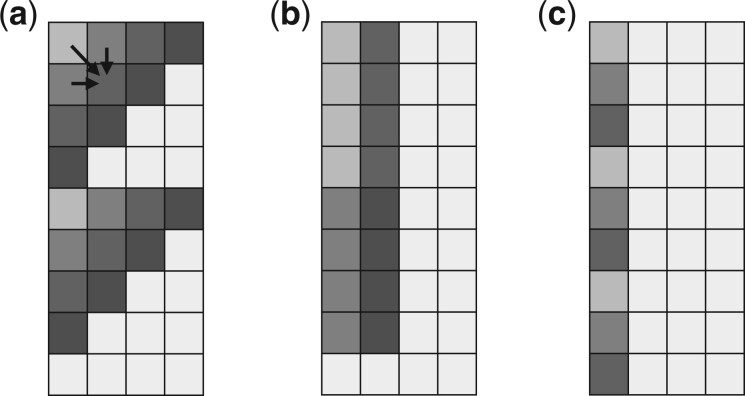
Vectorization strategies. Cells in the DP matrix with the same color can be computed in parallel with a single SIMD vector. (a) Vectors are laid out anti-diagonally. Arrows indicate DP cell dependencies. (b) Vectors are tiled vertically. Dependencies between adjacent cells within the same vector are typically resolved through prefix scan. (c) Striped layout where vectors hold noncontiguous cells within each column.

Since this alignment step is so critical, countless optimizations have been introduced in the past. Some recent advances has been on heuristics for avoiding or approximating full DP computation [e.g. with seeding or other means (Canzar and Salzberg 2017, Alser *et al.* 2021, Sahlin *et al.* 2022)], while other advances have made use of parallelism to speed up computing cells in the DP matrix for fine-grained alignments. These advances are complementary, and in many downstream applications, both approaches are used together for efficiency (Alser *et al.* 2021, Sahlin *et al.* 2022). In this article, we will focus on parallelizing DP computation. Many recent algorithms use Single Instruction Multiple Data (SIMD) instructions in modern CPUs to operate on multiple integers (lanes) in a wide (e.g. 128-bit or 256-bit) vector register in parallel. Here, we briefly review the most popular techniques for accelerating DP sequence alignment. In Fig. 1, we show the most popular methods for tiling SIMD vectors in the 2D DP matrix.

### 1.1 Bit parallel methods

For unit-cost edit distance computation, it is possible to pack differences between DP cells as bits in general-purpose registers (Myers 1999, Šošić and Šikić 2017). This can be generalized to handle integer scores (Loving *et al.* 2014). However, despite the high amount of parallelism achieved with bit vectors, these methods cannot handle complex, non-uniform scoring matrices.

### 1.2 Anti-diagonal methods

Since cells in the same anti-diagonal in the DP matrix do not depend on each other, anti-diagonal cells can be computed in parallel by tiling anti-diagonal SIMD vectors (Wozniak 1997). However, this approach makes it difficult to efficiently look up scores in a large scoring matrix, since SIMD memory gather operations are slow. (See here for a possible approach: https://github.com/eriksjolund/diagonalsw.)

### 1.3 Static banding

If the number of insertions or deletions can be upper bounded, then cells in the DP matrix can be pruned by not computing cells outside a (static) fixed-width band around the diagonal of the DP matrix (Ukkonen 1985). SIMD vectors are typically tiled anti-diagonally within this band (Wozniak 1997, Li 2018). However, this method cannot efficiently handle large gaps without massively increasing the band width. Note that it is possible to increase the band width exponentially if it is not known (Ukkonen 1985).

### 1.4 Adaptive banding

To achieve greater speedups over static banding, the anti-diagonal band can be made adaptive by greedily shifting it down and right based on the location of the best score (Suzuki and Kasahara 2017). Compared with the static banding approach that only computes DP cells around the main diagonal of the DP matrix, adaptive banding allows much smaller band widths to be used by allowing the band to adaptively stray from the main diagonal. To handle large scores that accumulate in long sequences without using wider SIMD lanes, the Suzuki–Kasahara difference recurrence algorithm (Suzuki and Kasahara 2018) introduced a method for storing the small (8-bit) differences between DP cells to maximize parallelism in SIMD vectors. The adaptive banding approach suffers from the greedy band shifting algorithm being unable to predict the correct shift direction when there are large gaps, causing it to return suboptimal alignment scores.

### 1.5 *X*-drop heuristic

The *X*-drop heuristic enables low scoring segments in an alignment to be avoided by terminating the alignment if the current alignment scores drop by *X* below the maximum score so far (Zhang *et al.* 2000). This can also be used to prune DP cells that drop by more than *X* below the max score, saving time (Altschul *et al.* 1997, Zhang *et al.* 2000, Suzuki and Kasahara 2017). Variants of this heuristic are used for efficiently extending seed matches in seed-and-extend approaches when aligning reads to a longer assembly (Sahlin *et al.* 2022).

### 1.6 Striped methods

Farrar’s (2007) algorithm is a SIMD-accelerated algorithm for computing the entire DP matrix. In contrast to anti-diagonal approaches, each SIMD vector holds scores from strided, noncontiguous cells in each column in the DP matrix. It requires an extra loop for lazily fixing up vertical dependencies. A similar algorithm is Daily’s prefix scan variant (Daily 2016), which also uses the striped pattern for storing cells. Since these striped methods compute the full DP matrix, they are suitable for both global and local alignment (Zhao *et al.* 2013) of sequences and PSSMs. However, these methods require the input sequences to be preprocessed in a striped fashion before DP for score lookups to be efficient during DP computation.

### 1.7 Wavefront alignment

The wavefront alignment (WFA) algorithm (Marco-Sola *et al.* 2021, 2022) uses a growing score threshold that iteratively increases while greedily expanding a wavefront of furthest reachable cells along the diagonals in the DP matrix. This method improves upon previous similar techniques (Ukkonen 1985, Myers 1986, 2014, Zhang *et al.* 2000) by using many practical optimizations (e.g. identifying exactly matching regions by packing multiple bytes in CPU registers), and massively reducing memory usage with BiWFA (Marco-Sola *et al.* 2022). However, this method cannot handle large scoring matrices efficiently and slows down for dissimilar sequences.

### 1.8 Intersequence parallelization

Although we focus on *intrasequence* parallelization techniques for pairwise sequence alignment in this article, there also exists methods for exploiting *intersequence* parallelization by aligning multiple sequences against a single sequence (Rognes 2011). This is done by storing the DP cells in multiple DP matrices for different sequences in a single SIMD vector. This is easier to parallelize, but the benefit of this approach only appears when aligning many input sequences of the same length.

Although CPUs are generally more easily available, alignment algorithms using GPUs [e.g. Logan (Zeni *et al.* 2020)] and other special devices also exist. It is also possible to align a sequence against a graph [e.g. abPOA (Gao *et al.* 2021)] or even aligning raw Nanopore signals (Gamaarachchi *et al.* 2020). However, they are out of the scope of this article as we focus on pairwise alignment on the CPU.

Despite these many recent advances in improving pairwise sequence alignment, they largely ignore challenges associated with accurately and efficiently handling position-specific scoring schemes, amino acid scoring schemes, and long alignment gaps in global, local, and *X*-drop alignment. In this article, we propose a new SIMD-accelerated algorithm for aligning sequences to other sequences or position-specific scoring matrices, called “Block Aligner.” We revisit ideas initially presented in adaptive banding (Suzuki and Kasahara 2017), which trades some accuracy in exchange for much greater efficiency through heuristics, but we expand upon it with our novel block shifting, growing, and shrinking architecture for adaptively aligning sequences. We show that our approach using blocks tiled with horizontal and vertical SIMD vectors makes efficiently handling complex scoring matrices possible. Finally, in our experiments, we show that Block Aligner performs well in practice on real protein and sequencing read datasets of various sequence lengths.

## 2 Methods

### 2.1 Smith–Waterman–Gotoh-like alignment

Here, we introduce a generalized version of the Smith–Waterman–Gotoh DP algorithm (Needleman and Wunsch 1970, Gotoh 1982) for aligning a sequence $q\in \Sigma^{\left|q\right|}$ over the alphabet Σ to a PSSM $p\in Z^{\left|p|\times |\Sigma \right|}$ with position-specific gap open/close penalties. Since at each position in the *p*, there is a score corresponding to each character in Σ, this is a generalization of the usual position-independent 20×20 amino acid or 4×4 nucleotide scoring matrices for sequence-to-sequence alignment. Hence, our presentation of the algorithm can be easily adapted for those use cases.

The generalized algorithm computes optimal alignment scores in a (|q|+1)×(|p|+1) matrix along with the transition directions (trace). In this article, we will use the convention of laying out *p* horizontally and *q* vertically (with one cell of padding at the beginning). For example, column *j* in the DP matrix corresponds to position j−1 in *p*. We use affine gap (insertion or deletion) penalties of the form $G_{\mathrm{ext}}\cdot g+G_{\mathrm{open}}^{r}[j]$ for transitions between different rows (gaps in *p*) and for transitions between different columns (gaps in *q*). We define $G_{\mathrm{ext}}$, $G_{\mathrm{open}}^{r}[j]$, $G_{\mathrm{open}}^{c}[j]$, and $G_{\mathrm{close}}^{c}[j]$, for each position 0≤j≤|p|, to be gap penalties, and *g* to be the length of a gap. For both *X*-drop and global alignment, the DP recurrence is: for 0≤i≤|q| and 0≤j≤|p|. Note that we use *D*, *R*, and *C* (*R* and *C* stand for “row” and “column”) in our notation instead of *H*, *E*, and *F* from the original Farrar paper (Farrar 2007).


$$G_{\mathrm{ext}}\cdot g+G_{\mathrm{open}}^{c}[j]+G_{\mathrm{close}}^{c}[j+g-1]$$

$$\begin{array}{c} C[i][j]=\max\{\begin{array}{c} C[i][j-1]+G_{\mathrm{ext}}\\ D[i][j-1]+G_{\mathrm{open}}^{c}[j]+G_{\mathrm{ext}} \end{array}\\ R[i][j]=\max\{\begin{array}{c} R[i-1][j]+G_{\mathrm{ext}}\\ D[i-1][j]+G_{\mathrm{open}}^{r}[j]+G_{\mathrm{ext}} \end{array}\\ D[i][j]=\max\{\begin{array}{c} D[i-1][j-1]+p[j-1][q[i-1]]\\ C[i][j]+G_{\mathrm{close}}^{c}[j]\\ R[i][j] \end{array}, \end{array}$$

For the boundary conditions, we have


$$\begin{array}{c} D[0][0]=0,\;C[i][0]=-\infty ,\;\text{and}\;R[0][j]=-\infty . \end{array}$$

Out of bounds cells or scores are set to −∞. In global alignment, the optimal score is D[|q|][|p|]. In *X*-drop alignment, it is the maximum computed *D* score.

### 2.2 Overview of Block Aligner

Block Aligner uses a novel block-based approach with tiled vertical or horizontal vectors in the DP matrix. We borrow ideas from adaptive banding (Suzuki and Kasahara 2017) to greedily shift a block right or down based on where the DP scores seem to be the largest. This adaptive approach allows a small block size to be used even if the optimal DP scores stray far from the diagonal in the DP matrix.

We also attempt to resolve a major limitation of previous adaptive approaches: large gaps and regions of mismatches easily fool the greedy block shifting algorithm. In Block Aligner, this issue is solved by allowing the block size to grow to span the erroneous region. Based on a heuristic similar to *X*-drop, Block Aligner can decide to dynamically recompute some regions in the DP matrix and double the block size. We use another heuristic to decide when to shrink the block size when there are less mismatches or gaps. Currently, we have implemented simple and efficient greedy heuristics based on the maximum DP score, but we expect that there are other potential heuristics that can be explored in future work.

Unlike the gapped BLAST *X*-drop algorithm (Altschul *et al.* 1997) that prunes cells only when they fail the *X*-drop test, Block Aligner is more optimistic: a smaller block size is assumed to start with, and it only grows when necessary.

### 2.3 The Block Aligner algorithm

**Figure 2.**
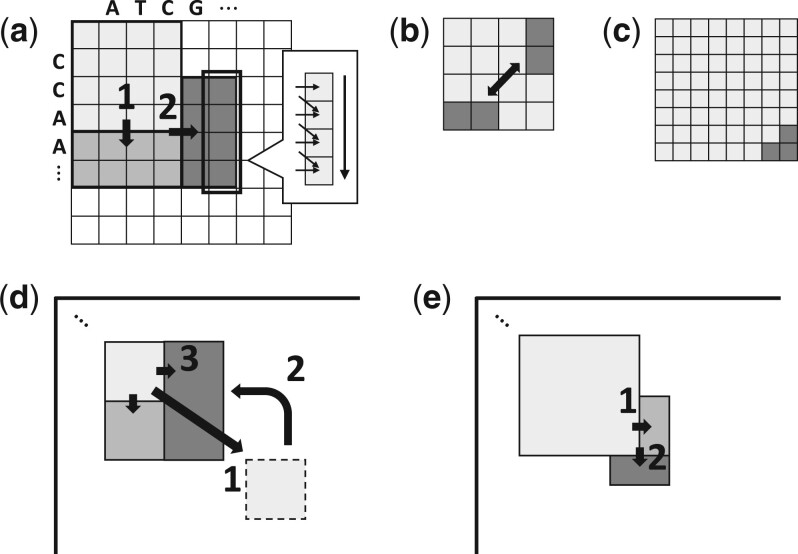
Diagram of the Block Aligner algorithm. (a) 1. Block shift down by a step size of S=2. 2. Block shift right. Each rectangular region is computed by tiling horizontal or vertical SIMD vectors. Prefix scan is used to resolve cell dependencies across a vector. (b) The max of the *S* cells in the bottom and right borders of a block of size B=4 are compared to determine the direction to shift. (c) Shrinking is determined by whether the max DP score occurs within the bottom-right corner cells (S=8 in this case). (d) 1. The block is shifted down and right. 2. When no new max score has been observed for multiple steps, some computed cells are discarded to return to a previous checkpoint state. 3. The block doubles in size and alignment restarts. (e) Block shrink followed by right shift and down shift.

**Figure 3.**
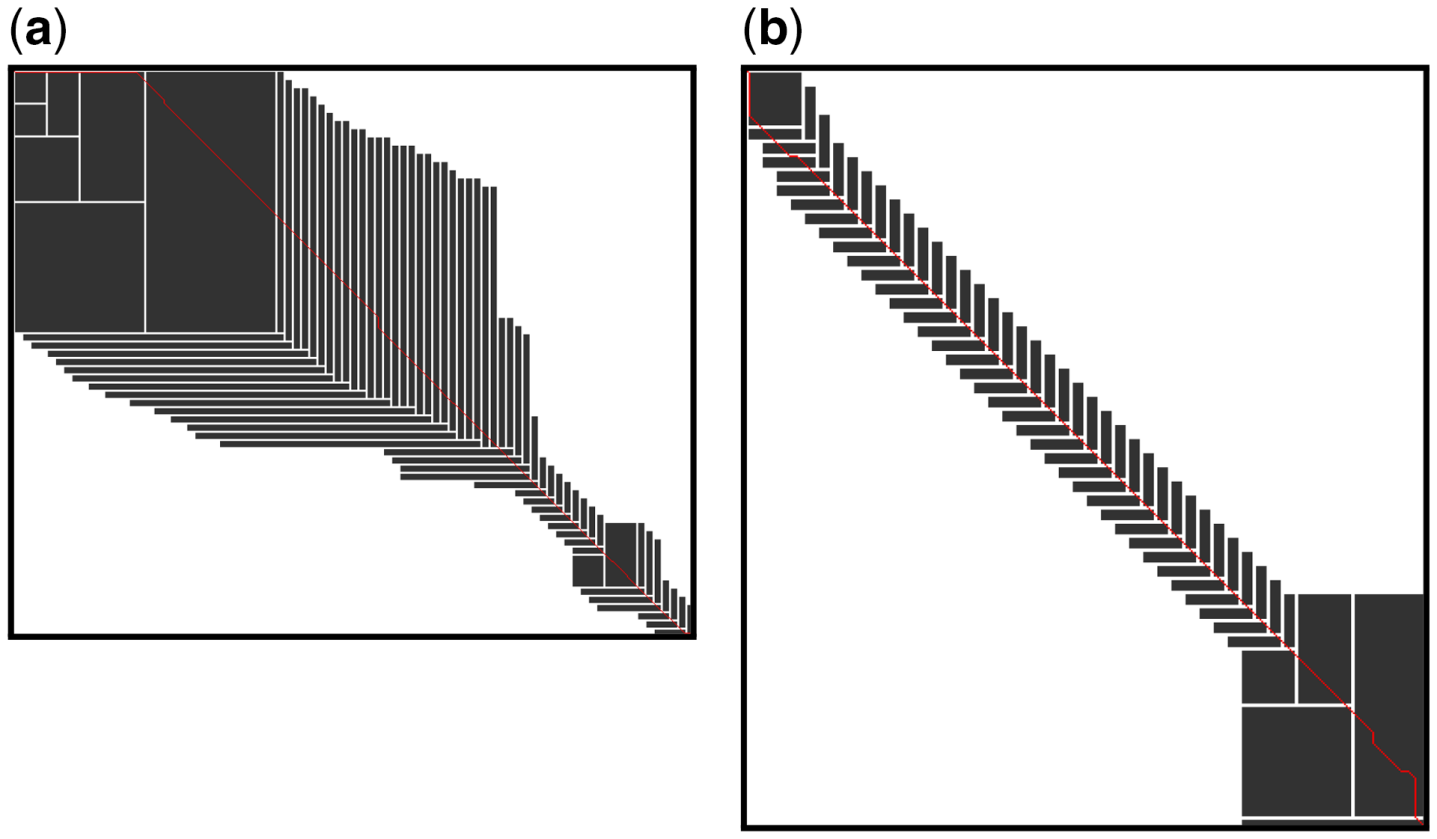
Block Aligner visualized. All regions computed by Block Aligner when aligning pairs of protein sequences from the Uniclust30 dataset are shaded. The red line stretching from the top left corner to the bottom right corner is the traceback path of the optimal alignment. Notice that Block Aligner generally tries to center the maximum scores (likely optimal alignment path) within the computed regions. Also, in (a), the block has to grow until it can span the initial gap and reach a series of matches that offset the cost of the gap. (a) Lower seq identity (54.9%) proteins. (b) Higher seq identity (72.3%) proteins.

We show pseudocode for our algorithm in Algorithm 1, which we use to guide our explanation of Block Aligner. We also give a diagram of Block Aligner in Fig. 2. To help visualize the cells computed by Block Aligner, we draw the computed rectangular regions for two pairs of real proteins in Fig. 3.

Algorithm 1. Pseudocode for the Block Aligner algorithm.

1: **procedure** ALIGN(*q*, *p*, *X*, $B_{\min}$, $B_{\max}$, $G_{\mathrm{open}}^{c}$, $G_{\mathrm{close}}^{c}$, $G_{\mathrm{open}}^{r}$, $G_{\mathrm{ext}}$)

2:  S←8, max_ score←0, dir←GROW, $B\leftarrow B_{\min}$, $B_{\mathrm{prev}}\leftarrow 0$, i←0, j←0

3:  Initialize *D*, $D^{\prime }$, *C*, *R*, and the trace array

4:  **while** true **do**                 ▹ One step of ligner. Note that *i*, *j*, *B*, and *dir* have already been updated.

5:   **if**dir=RIGHT**then**          ▹ Block position (i,j−S)↦(i,j)

6:    curr_ score←COMPUTE_RECT((i,j+B−S),(S,B))

7:   **else if**dir=DOWN**then**          ▹ Block position (i−S,j)↦(i,j)

8:    curr_ score←COMPUTE_RECT((i+B−S,j),(B,S))

9:   **else if**dir=GROW**then**

10:    $\mathrm{curr}\_\;\mathrm{score}\leftarrow \mathrm{COMPUTE}\_\mathrm{RECT}((i+B_{\mathrm{prev}},j),(B_{\mathrm{prev}},B-B_{\mathrm{prev}}))$          ▹ Grow down

11:    $\mathrm{curr}\_\;\mathrm{score}\leftarrow \mathrm{COMPUTE}\_\mathrm{RECT}((i,j+B_{\mathrm{prev}}),(B-B_{\mathrm{prev}},B))$          ▹ Grow right

12:   **end if**

13:

14:   **if**curr_ score>max_ score**then**

15:    Update max_ score with curr_ score and its store its position

16:    Save *i*, *j*, and the block right and bottom border scores as checkpoint

17:   **end if**

18:

19:   **if**curr_ score<max_ score−X for 2 consecutive steps, **then break**            ▹ X-drop

20:   **if**i+B>|q|**and**j+B>|p|, **then break**           ▹ Reached end of DP matrix

21:   **if**i+B>|q|**or**j+B>|p|, **then** shift *i*, *j* by *S* to avoid out of bounds and **continue**

22:

23:   **if**$B<B_{\max}$**and** *Y* steps pass without improving max_ score**then**           ▹ Block grow criteria

24:    $B_{\mathrm{prev}}=B$, B←2B, dir←GROW

25:    Restore *i*, *j*, and block border scores from previous checkpoint

26:    **continue**

27:   **end if**

28:

29:   **if**$B>B_{\min}$**and**max_ score is in the bottom-right corner region of block **then**           ▹ Block shrink criteria

30:    B←B/2, i←i+B, j←j+B

31:    Save *i*, *j*, and the block right and bottom border scores as checkpoint

32:   **end if**

33:

34:   dir←RIGHT or DOWN based on comparing right and bottom border cells

35:   Increment *i*, *j* by *S* based on *dir*

36:  **end while**

37:  **return** the trace array, and **if** *X*-drop, **then**max_ score, **else**D[|q|][|p|]

38: **end procedure**

#### 2.3.1 Computing scores

Block Aligner relies on the COMPUTE_RECT function in Algorithm 2 to efficiently compute the scores for certain rectangular regions of the DP matrix that has the top left corner at (r,c) and with dimensions (w,h). When shifting right [block at position (i,j−S) becomes (i,j)] or down [block at position (i−S,j) becomes (i,j)], a rectangular region of size S×B or B×S to the right or below the current block is computed, where *B* is the current block size and *S* is the step size (S<L≤B, S=8, and *L* is the number of 16-bit integers in a vector, so L=16 for AVX). When growing the block size from $B_{\mathrm{prev}}$ to $B=2B_{\mathrm{prev}}$, two rectangular regions must be computed: at the bottom [size $B_{\mathrm{prev}}\times (B-B_{\mathrm{prev}})$] and to the right of the current block [size $(B-B_{\mathrm{prev}})\times B$], in order to piece together a larger block at the same position. SIMD vectors need to be tiled vertically for right shift and horizontally for down shift. For simplicity, the COMPUTE_RECT function shown in Algorithm 2 is defined for right shifts, but it can be adapted for down shifts by swapping *R*, *C*, scoring matrices, and gap penalties. Note that DP cells that are not computed are treated as very large magnitude negative values.

Algorithm 2. Pseudocode for computing cells in a region of the DP matrix.

1: **procedure** COMPUTE_RECT((r,c), (w,h)) ▹ Right shift version

2:  i←r, j←c     ▹ Compute region [r,r+h)×[c,c+w)

3:  **while**j<c+w**do**

4:   **while**i<r+h**do**

5:    **for**$i^{\prime }\leftarrow 0$ to L−1**do**▹ Parallelized with SIMD in practice

6:     $C[i+i^{\prime }][j]\leftarrow \max\{\begin{array}{l} C[i+i^{\prime }][j-1]+G_{\mathrm{ext}}\\ D[i+i^{\prime }][j-1]+G_{\mathrm{open}}^{c}[j]+G_{\mathrm{ext}} \end{array}$

7:     $s\leftarrow p[j-1][q[i+i^{\prime }-1]]$▹ PSSM score lookup

8:     $D^{\prime }[i+i^{\prime }][j]\leftarrow \max\{\begin{array}{l} D[i+i^{\prime }-1][j-1]+s\\ C[i+i^{\prime }][j]+G_{\mathrm{close}}^{c}[j] \end{array}$

9:     $R^{\prime }\leftarrow \max_{-1\leq k<i^{\prime }}D^{\prime }[i+k][j]+(k+2)*G_{\mathrm{ext}}+G_{\mathrm{open}}^{r}[j]$     ▹ Max computed using prefix scan in practice

10:     $R[i+i^{\prime }][j]\leftarrow \max\{R^{\prime },R[i-1][j]+(i^{\prime }+1)*G_{\mathrm{ext}}\}$

11:     $D[i+i^{\prime }][j]\leftarrow \max\{D^{\prime }[i+i^{\prime }][j],R[i+i^{\prime }][j]\}$

12:     Keep track of transition type in trace array

13:     Keep track of the maximum computed *D* score

14:    **end for**

15:     i←i+L

16:   **end while**

17:   j←j+1

18:  **end while**

19:  **return** maximum computed *D* score and its position

20: **end procedure**

We tile vectors vertically and horizontally, resolving dependencies between lanes within the SIMD vectors (computing the $R^{\prime }$ array) using prefix scan, an O(log L) time operation. This has been explored previously in Suzuki and Kasahara (2017); Gao *et al.* (2021) on CPU and in Khajeh-Saeed *et al.* (2010) on GPU. However, our implementation has a couple of important optimizations. For 256-bit AVX vectors, we avoid the performance penalty for moving values between its lower and higher 128-bit lanes by performing prefix scan on the 128-bit lanes separately (Kogge and Stone 1973), then doing one lane-crossing shuffle to resolve the dependency between the upper and lower halves of SIMD vector (Sklansky 1960). Additionally, we reorder the dependency of $R[i+i^{\prime }][j]$ on the R[i−1][j] value of the previous vector to be after the prefix scan step. This allows some instruction-level parallelism to be exploited, since prefix scans for computing $R^{\prime }$ from multiple iterations of the loop can be computed independently, and afterward, the dependencies between vectors can be resolved by updating the current iteration’s $R^{\prime }$ with the last *R* value of the previous iteration (see Supplementary Materials for more information). Similar ideas have been explored in the context of GPU prefix scans (Merrill and Garland 2016).

We do not tile vectors anti-diagonally (Wozniak 1997) because it makes looking up large scoring matrices for multiple lanes in the SIMD vector difficult. Loading scores for a horizontal or vertical vector in the DP matrix is much easier: part of a single row or column of the PSSM or 20×20 amino acid scoring matrix is loaded, and the scores for each cell in the SIMD vector can be determined through fast vector shuffles (e.g. a combination of _mm256_shuffle_epi8 and _mm256_blendv_epi8 to lookup a 32-element row in the score matrix with a 5-bit index).

We do not use the striped profile in Farrar’s (2007) algorithm and Daily’s (2016) prefix scan variant because they require precomputed striped profiles for the query sequence, which is not possible to obtain when the computed block shifts dynamically.

#### 2.3.2 Score offsets

Although not shown in the pseudocode, we use 16-bit lanes in SIMD vectors to represent scores relative to a 32-bit offset for each block. We choose the maximum score from the previous step as the current step’s offset to ensure that larger scores are accurately represented, even if they exceed 16 bits. Since we cannot use the difference recurrence method (Suzuki and Kasahara 2018) along with prefix scans, we were unable to use narrow 8-bit lanes for more parallelism.

#### 2.3.3 Determining shift direction

To determine the direction to shift the block in the next step, we compare the max of the *S* leftmost scores in the bottom border with the max of the *S* topmost scores in the right border:


$$\mathrm{dir}=\{\begin{array}{cc} \mathrm{RIGHT},\; & \text{if}\;\underset{k=0\ldots S-1}{\max}D[i+k][j+B-1]\\ & \;\geq \underset{k=0\ldots S-1}{\max}D[i+B-1][j+k]\\ \mathrm{DOWN},\; & \text{otherwise} \end{array}.$$

Our method differs from locating the position of the max score in an adaptive band (https://github.com/giuliaguidi/XAVIER) or comparing only the bottom-left and top-right corner cells in an adaptive band (Suzuki and Kasahara 2017). In preliminary experiments, we found that our method strikes a good balance between speed, simplicity, and accuracy.

#### 2.3.4 Block growing/shrinking and restoring checkpoints

In order to handle large gaps, we save checkpoints and use a heuristic to determine when there is a gap and the block size needs to grow. Every time a new maximum score is encountered, a checkpoint of the current state of the block is saved. If, after some threshold *Y* number of steps, a new maximum score has not been reached, then the block returns to the previous checkpoint and the block size grows. The block size is allowed to repeatedly grow in quick succession if a new max score is not identified after growing. Any regions that were computed since the last checkpoint are discarded when restoring the checkpoint. Note that only the bottom-most row and rightmost column of a block needs to be stored for both the current state and the previous checkpoint state, so the space complexity is O(B).

The *Y* threshold is similar to the *X*-drop threshold, but it represents the number of steps, which is scoring-matrix-agnostic. We fix Y=⌊B/S⌋−1, which means that the *Y* threshold is met if the block shifts approximately *B* cells away from where the last maximum score is encountered.

If the current max score is in the S/4 rightmost cells of the bottom border or the S/4 bottom-most cells of the right border of the block, then the block size is halved. A block at position (i,j) with size *B* in the DP matrix will become a block at position (i+B/2,j+B/2) with size B/2. This restrictive criteria for shrinking ensures that the max score, and hence the optimal alignment path, is highly likely to be centered within the smaller block.

#### 2.3.5 Traceback

To obtain the exact edit operations used, we use the movemask instruction (e.g. _mm256_movemask_epi8) to compress the DP matrix’s trace direction information and only store 4 bits per DP cell: 2 bits for direction (diagonal, right, or down) and 2 bits for opening or extending gaps in *R* and *C*. The trace data are treated like a stack: when restoring a previous checkpoint, trace information for discarded blocks are popped off until the checkpoint is reached. The memory complexity for sorting trace directions is $O(B_{\max}(|p|+|q|))$. If the traceback path (e.g. CIGAR) is needed, the stored 4-bit encoded trace directions can be decoded starting from the end of the alignment and going back to the origin by using a lookup-table-based state machine.

### 2.4 Library implementation

Block Aligner is implemented as a Rust library. Currently, Block Aligner supports x86 SSE2/AVX2, ARM Neon, and Webassembly (Haas *et al.* 2017) SIMD instruction sets. C bindings are also available for Block Aligner. Due to Block Aligner’s heavy usage of unsafe SIMD operations, we have carefully tested it with both manually specified test cases and simulated sequences. We have profiled and carefully examined the compiled assembly code of the core Block Aligner implementation to optimize its performance.

## 3 Results

### 3.1 Setup

We evaluate Block Aligner on multiple challenging datasets of wildly varying sequence lengths and similarity:

- Illumina reads and 1 kbp, <10 kbp, and <50 kbp Nanopore reads. We use DNA read datasets from Marco-Sola *et al.* (2021, 2022), where the sequence pairs are derived from minimap2 (Li 2018) alignments. The Illumina short read dataset is 100 000 pairs of sequences sampled from Illumina HiSeq 2000 reads of length 101, with accession number ERX069505. The 1 kbp, <10 kbp, and <50 kbp Nanopore long read datasets have 12 477, 5000, and 10 000 pairs of sequences, respectively, and they are sampled from Oxford Nanopore MinION reads from Bowden *et al.* (2019), with accession numbers ERR3278877 to ERR3278886. The Nanopore sequence pairs have an average sequence identity of around 90%. The Illumina and Nanopore sequence pairs contain differences that represent both sequencing errors and genetic variation.
- Uniclust30. We use mmseqs2 (Steinegger and Söding 2017) to find pairs of alignable sequences above a certain coverage threshold (percent overlap between two sequences) in the Uniclust30 (Mirdita *et al.* 2017) (UniRef30 version 03_2021) database. Two coverage thresholds are used: 80%, which is the mmseqs2 default, and 95%, which is an easier dataset that is more globally alignable. We will refer to these datasets with different coverage thresholds as “uc30” and “uc30 0.95,” respectively. For both datasets, the proteins range from between around 20 to around 8000 amino acids long, with an average length of around 300. There are 7000 protein pairs per dataset.
- SCOP domain PSSMs. We use mmseqs2 (Steinegger and Söding 2017) to search for protein domains in the SCOPe 2.01 database (Fox *et al.* 2014) that are similar to each SCOP domain. Note that sequences in this database are <40% identity with each other. The search results are used to build position-specific scoring matrices (no position-specific gap penalties). Then, we create a dataset of 11 160 pairs of sequences and PSSMs to align by randomly choosing protein domains and position-specific scoring matrices from the same SCOP family. The lengths of the protein domains range from 5 to around 1400, with an average length of around 174. Protein domains are globally alignable and they are shorter than full protein sequences.

For ground truth global alignment scores with full DP, we use the scalar DP algorithm in Rust-bio (Köster 2016) and Parasail’s implementation of Farrar’s (2007) striped SIMD algorithm. We mainly focus on evaluating global alignment because it is easier to directly compare alignment scores. For performance benchmarks on proteins, we evaluate Block Aligner against Parasail (Daily 2016)’s implementation of Farrar’s (2007) algorithm. This implementation will attempt to align with 8-bit SIMD lanes for speed, and only fall back to 16-bit lanes if an overflow is detected. For performance benchmarks on DNA sequences, we evaluate Block Aligner against Edlib (Šošić and Šikić 2017), ksw2 (Li 2018), Parasail’s implementation of Farrar’s striped SIMD algorithm, and WFA (Marco-Sola *et al.* 2021) via WFA2-lib (https://github.com/smarco/WFA2-lib).

When measuring the accuracy or error rate of an algorithm, we typically count how many pairs of alignments are suboptimal compared with the true score from full DP. To measure how much a predicted score differs from the true score, we compute the percent error, which is


$$\%\;\text{error}=\frac{\text{true}\;\text{score}-\text{pred}\;\text{score}}{\text{true }\;\text{score}}.$$

We will write $B_{\min}–B_{\max}$ (e.g. 32–256) to indicate the min and max block sizes. We use the BLOSUM62 (Henikoff and Henikoff 1992) scoring matrix with fixed gap penalties ($G_{\mathrm{open}}$ = −10, $G_{\mathrm{ext}}$ = −1, no $G_{\mathrm{close}}$) for proteins and minimap2 scores (match = 2, mismatch = −4, $G_{\mathrm{open}}$ = −4, $G_{\mathrm{ext}}$ = −2) for DNA sequences (Li 2018). We use costs (match = 0, mismatch = 4, $G_{\mathrm{open}}$ = 4, $G_{\mathrm{ext}}$ = 2) when aligning with WFA2-lib. We will refer to the wf-adaptive heuristic (Marco-Sola *et al.* 2021), with parameters (10, 1%, 1), as “wfa2 adaptive” in our experiments (the threshold is set to 1% of sequence length for speed). Edlib only supports edit distance (unit costs) and it uses exponential search on the band width.

We will often refer to the sequence identity of a pair of sequences, which is defined as


$$\text{seq}\;\text{id}=\frac{\#\;\text{matches}}{\#\;\text{matches}+\#\;\text{mismatches}+\#\;\text{insertions}+\#\;\text{deletions}}.$$

All of our benchmarks are conducted on a 2019 MacBook Pro with 2.3 GHz Intel Core i9 CPUs and 16 GB of RAM. Our benchmarks use 256-bit AVX2 vector registers. The run times reported are for the whole dataset. The code, data, and steps to reproduce our experiments are available on Github (https://github.com/Daniel-Liu-c0deb0t/block-aligner).

### 3.2 Sequencing reads benchmark

**Figure 4.**
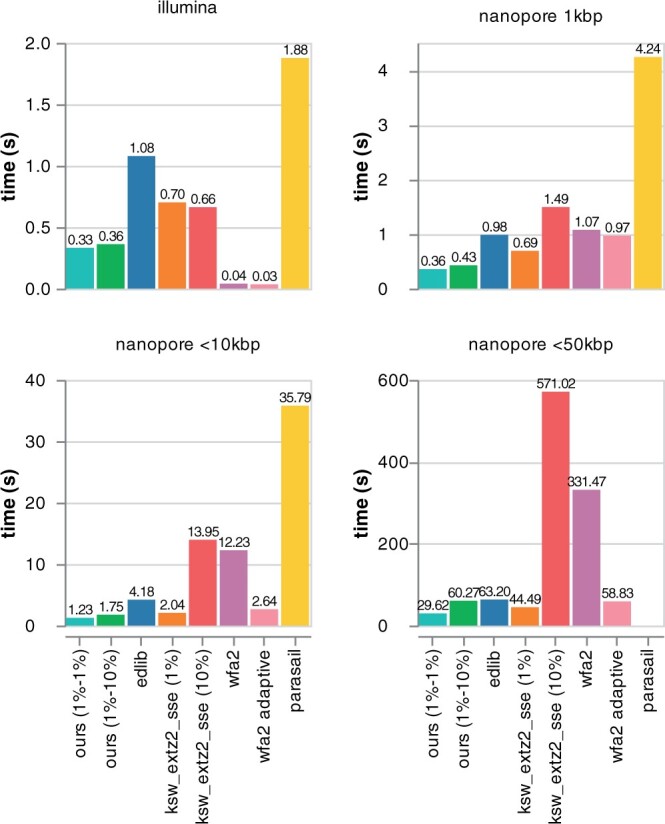
Speed of global alignment of datasets of DNA reads for different algorithms. Tracebacks are computed for all algorithms except Parasail. Block sizes and ksw2’s static band widths are percentages of sequence lengths. Algorithms that run for too long are not shown. Note that the *y*-axis is scaled differently for each plot.

We evaluate the global alignment of Illumina and Oxford Nanopore reads ranging from around 100 bp to 50 kbp with Block Aligner. The accuracy results are shown in Table 1 and the benchmark results are shown in Fig. 4. We found that for the Nanopore datasets, setting the block size to range from around 1% to 10% of the sequence length (with block sizes being at least 32) resulted in less than 3% error rate overall, while running two times faster than Edlib for short sequences and around six times faster than WFA on erroneous Oxford Nanopore reads. As expected, for accurate Illumina short reads, WFA is much faster than Block Aligner. Compared with WFA adaptive, Block Aligner’s error rate is nearly five times lower for <10 kbp Oxford Nanopore reads (see Supplementary Materials). We also benchmark Block Aligner without block growing (same min and max block sizes) and ksw2 with band width equal to the min block size to show that the performance penalty of block growing/shrinking is minimal, especially when compared to ksw2 with a static band width of 10%. Additionally, with block growing/shrinking, we observe that Block Aligner correctly aligns reads with gaps up to 3400 bp, which is much larger than the min block size (see Supplementary Materials for details). However, note that without block growing, Block Aligner’s error rate reaches up to 30%, highlighting the importance of our heuristics.

**Table 1.** Error rate of gBlock Alilobal alignment of DNA reads with Block Aligner.

Dataset	Read pairs	Errors	Error rate (%)	% Error
Illumina	100 000	0	0.0	0.0
Nanopore 1 kbp	12 477	21	0.2	6.0
Nanopore <10 kbp	5000	109	2.2	3.6
Nanopore <50 kbp	10 000	278	2.8	2.1

*Notes*: The scores produced by Block Aligner are compared to full DP global alignment. Block sizes of 1%–10% of sequence lengths are used. % error is defined as the average percent error over Block Aligner scores that are not optimal.

**Table 2.** Global alignment of protein domains to position-specific scoring matrices derived from SCOP domains.

Algorithm	Block size	Error rate (%)	Run time (s)
Ours	32–32	13.5	0.15
Ours	32–64	3.9	0.18
Ours	32–128	0.7	0.21
Ours	128–128	0.5	0.21
Parasail	–	–	0.60

*Notes*: True scores are computed by aligning with Block Aligner with a block size of 2048–2048. Block Aligner computes tracebacks and Parasail computes sequence to PSSM consensus sequence alignment.

### 3.3 Uniclust30 protein benchmark

**Figure 5.**
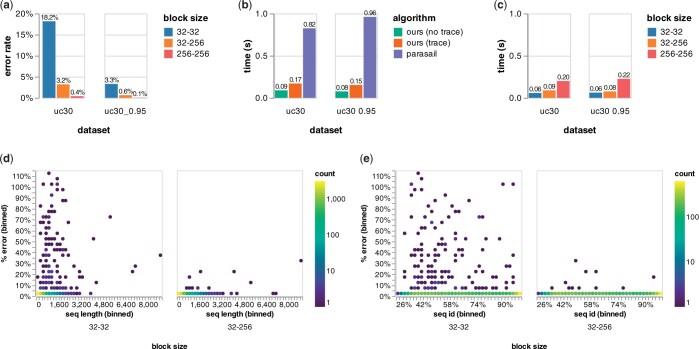
Global alignment of proteins from the Uniclust30 dataset with Block Aligner. Panels (d) and (e) are based on the “uc30 0.95” dataset. Parasail is used to only compute the score. (a) Error rate of Block Aligner for different block sizes. (b) Speed of Block Aligner (block size 32–256) compared to Parasail. (c) Speed of Block Aligner for different block sizes (no trace). (d) Percent error of Block Aligner scores compared to true scores for different sequence lengths. (e) Percent error of Block Aligner scores compared to true scores for different sequence identities.

We align pairs of proteins from the Uniclust30 dataset (Mirdita *et al.* 2017) and report our results in Fig. 5. It is clear from Fig. 5a and c that allowing the block size to grow/shrink in the range 32–256 results in accuracy that is similar to a fixed block size of 256, but speed that is similar to a fixed block size of 32. In other words, our algorithm provides the best of both worlds, with its heuristics and well-optimized SIMD DP and prefix scan implementation. In Fig. 5b, we see that compared with Parasail (Daily 2016), Block Aligner with a block size of 32–256 is around 9–12 times faster for score only. Block Aligner is also easy to use since we did not need to tune any parameters, other than providing a block size range, to handle proteins of wildly different sequence identities and lengths (Fig. 5d and e).

### 3.4 SCOP protein domains benchmark

In Table 2, we show the performance of Block Aligner for aligning proteins domains to PSSMs in the same SCOP families. These results are consistent with Uniclust30 results, where allowing the block size to grow improves accuracy. However, the performance improvement of block growing/shrinking is small, since the domains/PSSMs are shorter than full length proteins and the sequence identity between domains is low.

## 4 Discussion

Although Block Aligner generates some suboptimal alignments, our method enables efficient alignment computation with generalized scoring schemes and varying sequence lengths/identities. In downstream applications, Block Aligner provides a faster alternative to WFA (Marco-Sola *et al.* 2021) for aligning divergent sequences, and Farrar’s (2007) algorithm for protein sequences and PSSM profiles (Zhao *et al.* 2013, Daily 2016, Steinegger and Söding 2017). For example, Block Aligner has been adapted to align proteins with both amino acid sequences and 3Di sequences that encode amino acid interactions in Foldseek (van Kempen *et al.* 2023). Another potential application of Block Aligner is quickly computing a lower bound on the alignment score by purposefully constraining the block size. This can be useful for filtering sequences before more stringent alignment. On the other hand, if greater accuracy is desired, then larger minimum block sizes can be used, though at the expense of speed.

For future work, Block Aligner can be extended to support other SIMD instruction sets (e.g. AVX-512), GPUs, and ultra-long reads (>100 kbp long). Finally, although our results show that simple greedy heuristics work well, there is room to explore additional heuristics [e.g. seeding to guide alignment (Groot Koerkamp and Ivanov 2022)] and tradeoffs in the time spent on SIMD DP computation versus heuristics.

## 5 Conclusion

Since alignment is a core computational primitive in bioinformatics, it is important to explore the tradeoff between efficiency, flexibility, and accuracy of alignment algorithms. We believe Block Aligner will enable more efficient analysis of large protein and sequencing datasets, with minimal accuracy loss.

## Supplementary Material

btad487_Supplementary_Data

## Data Availability

The data underlying this article are available on Github at https://github.com/Daniel-Liu-c0deb0t/block-aligner/blob/main/data/README.md.
