# Supplementary material for: Block Aligner: an adaptive SIMD-accelerated aligner for sequences and position-specific scoring matrices
Source: Bioinformatics. 2023 Aug 3;39(8):btad487. doi: 10.1093/bioinformatics/btad487 (PMC10457662; doi:10.1093/bioinformatics/btad487)

Supplementary Materials for Block Aligner: an adaptive SIMD-accelerated aligner for sequences and position-specific scoring matrices

Daniel Liu and Martin Steinegger

S1 Prefix scan

In Figure S1, we show a simplified version of our prefix scan implementation for resolving dependencies between DP cells.

S2 Varying scoring parameters

We show the error rate of Block Aligner with a few different scoring methods on <10kbp Nanopore reads in Table S1. Note that (match = 2, mismatch = -4, G_{open} = -4, G_{ext} = -2) are the default minimap2 scoring parameters. Note that when specifying gap penalty parameters for Block Aligner for protein and DNA sequences, the gap open penalty parameter should be set to $G_{open} + G_{ext}$. This slight implementation difference helps avoid having to do an extra addition in the DP computation. We see that with higher penalties, the error rates of Block Aligner and WFA2 adaptive decrease.

Table S1: Error rate of Block Aligner for different scoring parameters on <10kbp Nanopore reads. Block sizes are percentages of sequence lengths are used. The error rate of WFA2 (WFA2-lib) adaptive with parameters (10, 1%, 1) is also shown (1% is computed based on sequence lengths). WFA2 adaptive is run with the same scoring parameters in the table, except with match score equal to 0.

	scores			error rate		
match	mismatch	G_{open}	G_{ext}	ours (1%-10%)	ours (1%-1%)	WFA2 adaptive
1	-1	-1	-1	4.2%	32.6%	29.0%
2	-4	-4	-2	2.2%	31.9%	10.2%
2	-4	-8	-2	1.8%	31.5%	7.8%

Figure S1: Visualization of prefix scan. In (a), the rows represent successive steps of prefix scan in an 128-bit lane (8 16-bit integers). The last (bottom) row is the result. The number in each cell indicates the number of cells to the left (including itself) that contributes to this cell. Arrows indicate the flow of data. The vector is repeatedly shifted by powers of two to resolve all dependencies. In (b), dependencies between between the upper and lower 128-bit halves of the 256-bit AVX vector register are resolved by using the last cell of the lower 128-bit half. Finally, the dependencies between the previous 256-bit vector and the current vector is resolved by using the last cell of the previous vector. This construction allows prefix scans to be computed efficiently for any number of cells.

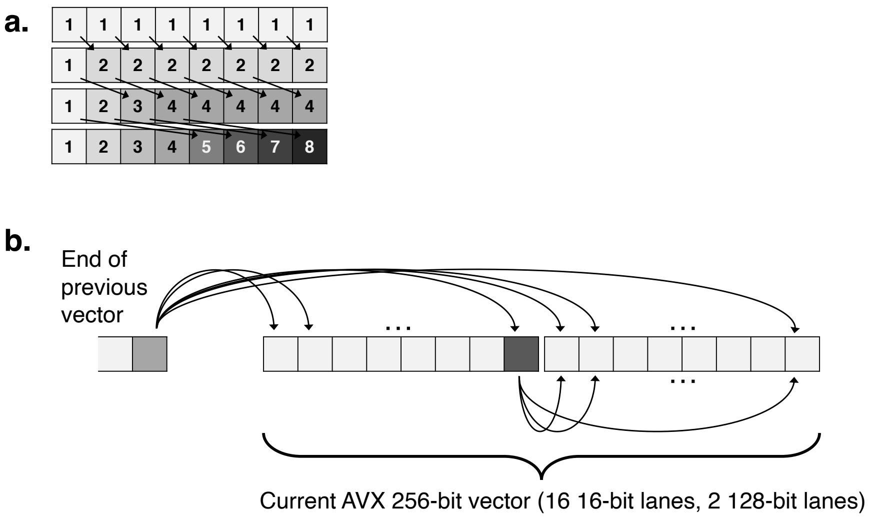

S3 Large gaps in alignments

In Figure S2, we show how Block Aligner performs for sequences with different largest gap (indel) lengths in their alignment. Since Block Aligner is accurate even for gaps much greater than the min block size (1% of sequence length), this shows that the block growing heuristics are very helpful.

Figure S2: % error of Block Aligner alignment scores for sequences with different largest gap (indel) lengths. Block sizes of 1%-10% of sequence lengths are used.

(a) <10kbp Nanopore reads. **(b)** <50kbp Nanopore reads.

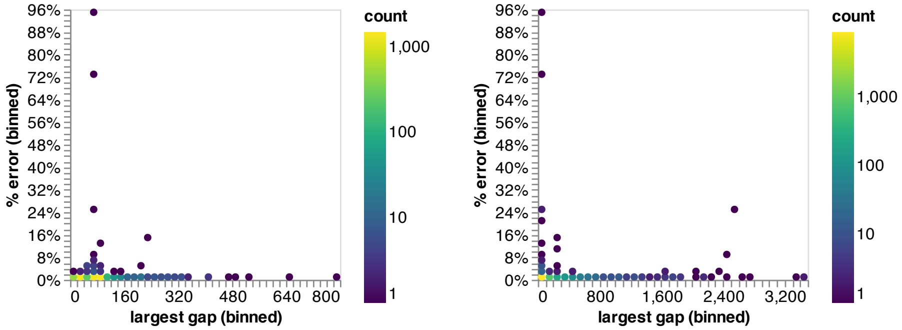

S4 Comparing alignment scores

In Figure S3, Figure S4, and Figure S5, we show how Block Aligner's scores compare to the true scores for global alignment on a variety of datasets. Note that Block Aligner scores could be suboptimal and lower than the true score. We expect alignments with lower true scores to more likely be errors, due to the higher sequence divergence.

S5 Rust bindings

In our benchmarks against other alignment algorithms, we used Rust bindings for Parasail (`https://github.com/Daniel-Liu-c0deb0t/parasailors-new`), Edlib (`https://github.com/Daniel-Liu-c0deb0t/edlib-rs`), ksw2 (`https://github.com/pairwise-alignment/ksw2-sys`), and WFA2-lib (`https://github.com/pairwise-alignment/rust-wfa2`). These bindings should have negligible overhead.

Figure S3: Block Aligner predicted scores compared to true scores for Nanopore reads. Block sizes of 1%-10% of sequence lengths are used.

(a) <10kbp Nanopore reads.

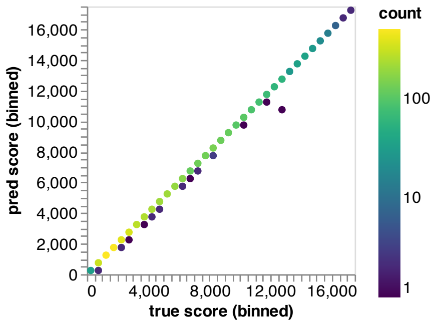

(b) <50kbp Nanopore reads.

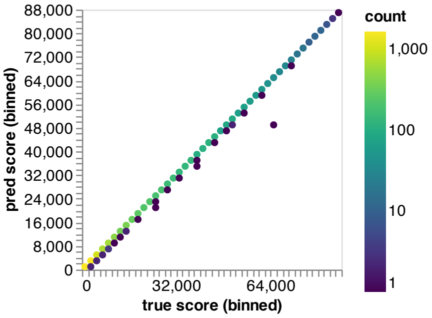

Figure S4: Block Aligner predicted scores compared to true scores for Uniclust30 proteins.

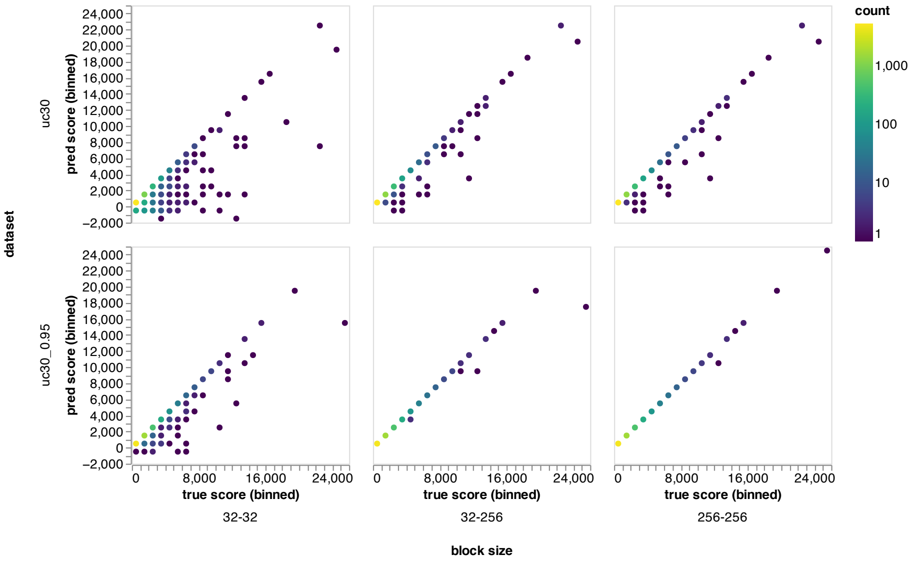

Figure S5: Block Aligner predicted scores compared to true scores for SCOP domains.

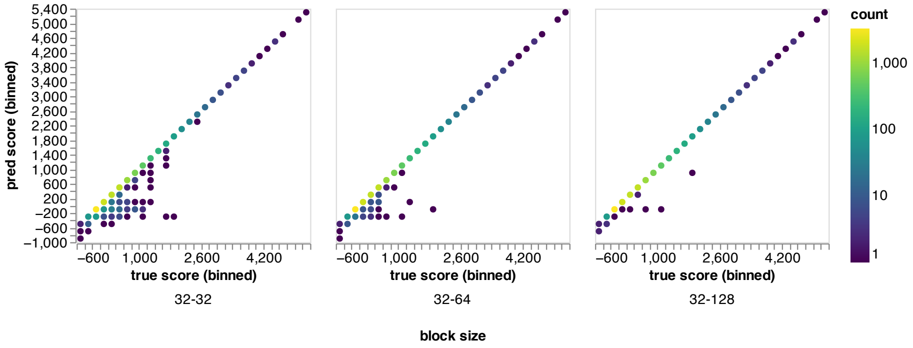

Supplement: btad487_Supplementary_Data [file btad487_supplementary_data.pdf]
